# Altered resting-state functional connectivity of the cerebellum in schizophrenia

**DOI:** 10.1007/s11682-017-9704-0

**Published:** 2017-03-14

**Authors:** Chuanjun Zhuo, Chunli Wang, Lina Wang, Xinyu Guo, Qingying Xu, Yanyan Liu, Jiajia Zhu

**Affiliations:** 1 Department of Radiology and Tianjin Key Laboratory of Functional Imaging, Tianjin Medical University General Hospital, No. 154, Anshan Road, Heping District, Tianjin, 300052 China; 2 Department of Psychiatry, Wenzhou Seventh People’s Hospital, Wenzhou, Zhejiang Province, 325000 China; 3 Tianjin Mental Health Center, Tianjin Anding Hospital, Tianjin, 300222 China; 4 Tianjin Anning Hospital, Tianjin, 300300 China; 5 Department of Radiology, The First Affiliated Hospital of Anhui Medical University, Hefei, 230022 China

**Keywords:** Schizophrenia, Cerebellum, Resting-state fMRI, Functional connectivity, Functional connectivity density

## Abstract

Structural and functional abnormalities of the cerebellum in schizophrenia have been reported. Most previous studies investigating resting-state functional connectivity (rsFC) have relied on a priori restrictions on seed regions or specific networks, which may bias observations. In this study, we aimed to elicit the connectivity alterations of the cerebellum in schizophrenia in a hypothesis-free approach. Ninety-five schizophrenia patients and 93 sex- and age-matched healthy controls underwent resting-state functional magnetic resonance imaging (fMRI). A voxel-wise data-driven method, resting-state functional connectivity density (rsFCD), was used to investigate cerebellar connectivity changes in schizophrenia patients. Regions with altered rsFCD were chosen as seeds to perform seed-based resting-state functional connectivity (rsFC) analyses. We found that schizophrenia patients exhibited decreased rsFCD in the right hemispheric VI; moreover, this cerebellar region showed increased rsFC with the prefrontal cortex and subcortical nuclei and decreased rsFC with the visual cortex and sensorimotor cortex. In addition, some rsFC changes were associated with positive symptoms. These findings suggest that abnormalities of the cerebellar hub and cerebellar-subcortical-cortical loop may be the underlying mechanisms of schizophrenia.

## Introduction

Although traditionally associated with motor function (Paulin 1993), the cerebellum is now seen to also be involved in both cognitive and affective functions (Gordon 2007; Schmahmann and Sherman 1998). The involvement of the cerebellum in these functions may be related to its connection to several functionally heterogeneous cortical and subcortical regions through a cerebellar-subcortical-cortical loop. Until now, the converging lines of evidence point toward structural and functional abnormalities of the cerebellum in schizophrenia (Andreasen and Pierson 2008; Lungu et al. 2013; Picard et al. 2008). For example, schizophrenia patients have exhibited altered cerebellar gray and white matter volume (Kuhn et al. 2012; Laidi et al. 2015; Lee et al. 2007), abnormal task-related activation of the cerebellum (Bernard and Mittal 2015), and cerebellar functional and anatomical connectivity abnormalities (Collin et al. 2011; Liu et al. 2011; Wang et al. 2014a). Moreover, several cerebellar abnormalities have been associated with neurological soft signs (Bottmer et al. 2005; Hirjak et al. 2015; Thomann et al. 2009), psychotic symptoms (Garg et al. 2013; Ichimiya et al. 2001), and cognitive deficits (Lee et al. 2007; Okugawa et al. 2007; Segarra et al. 2008) in schizophrenia.

Seed-based or independent component analysis (ICA) approaches measuring resting-state functional connectivity (rsFC) have relied on a priori restrictions on seed regions or specific networks, which may bias observations. Recently, resting-state functional connectivity density (rsFCD) analysis has been developed to construct whole-brain functional connectivity networks based on resting-state functional magnetic resonance imaging (fMRI) datasets (Tomasi and Volkow 2010, 2011a, b). This voxel-wise data-driven method might provide an unbiased approach to analyze whole-brain connectivity by measuring the temporal correlations of every pair of voxels in the entire brain. rsFCD is also referred to as the nodal degree centrality of binary networks in graph theory (Buckner et al. 2009), and the brain regions with higher rsFCD are considered functional hubs that play a more important role in the information processing of the whole brain than those with lower rsFCD. This powerful method has been applied to investigate alterations of the distribution of cerebral hubs in schizophrenia (Chen et al. 2015; Guo et al. 2015; Wang et al. 2014b; Zhuo et al. 2014); however, alterations in rsFCD of the cerebellum in schizophrenia remain largely unknown.

In the present study, resting-state fMRI data were collected from 95 schizophrenia patients and 93 healthy controls. Inter-group differences in rsFCD and rsFC of the cerebellum and their relationships with clinical variables were investigated.

## Materials and methods

### Subjects

Two hundred right-handed individuals were enrolled in this study, including 98 schizophrenia patients and 102 healthy controls. The Medical Research Ethics Committee of Tianjin Medical University General Hospital approved this study. After a complete description of the study, written informed consent was obtained from each subject. The diagnosis of schizophrenia was determined by the consensus of two experienced clinical psychiatrists using the Structured Interview for DSM-IV Axis I Disorders, Patient Edition (SCID-P). Healthy controls were recruited from the local community via advertisements. All healthy controls were screened using the non-patient edition of the SCID (SCID-NP) to confirm a lifetime absence of psychiatric illnesses. In addition, all of the healthy controls were interviewed to exclude individuals with a known history of psychiatric illness in first-degree relatives. The exclusion criteria for all subjects were MRI contraindications, a history of head trauma with consciousness disturbances lasting more than five minutes, a history of drug or alcohol abuse, pregnancy, and any physical illness such as cardiovascular disease or neurological disorders, as diagnosed by an interview and medical records review. A professional radiologist assessed the image quality slice-by-slice, and three patients and 9 healthy controls with poor image quality were excluded. Consequently, 95 schizophrenia patients and 93 healthy controls were included in the statistical analysis. The clinical symptoms of psychosis were quantified using the Positive and Negative Syndrome Scale (PANSS). The antipsychotic dosages are reported as the chlorpromazine equivalents calculated based on clinically equivalent dosing estimates (Gardner et al. 2010). For each schizophrenia patient, the chlorpromazine equivalent was estimated according to the antipsychotic drugs and dosages used in the latest week before MRI.

### MRI data acquisition

MRI data were acquired using a 3.0-Tesla MR system (Discovery MR750, General Electric, Milwaukee, WI, USA). Tight but comfortable foam padding was used to minimize head motion, and earplugs were used to reduce scanner noise. Sagittal 3D T1-weighted images were acquired using a brain volume sequence and the following parameters: repetition time (TR) = 8.2 ms; echo time (TE) = 3.2 ms; inversion time (TI) = 450 ms; flip angle (FA) = 12°; field of view (FOV) = 256 mm × 256 mm; matrix =256 × 256; slice thickness = 1 mm, no gap; and 188 sagittal slices. Resting-state fMRI data were acquired using a gradient-echo single-short echo planar imaging sequence with the following parameters: TR/TE = 2000/45 ms; FOV = 220 mm × 220 mm; matrix =64 × 64; FA = 90°; slice thickness = 4 mm; gap =0.5 mm; 32 interleaved transverse slices; and 180 volumes. All subjects were instructed to keep their eyes closed, relax, move as little as possible, think of nothing in particular, and not fall asleep during the fMRI scans.

### fMRI data preprocessing

Resting-state fMRI data were preprocessed using SPM8 (http://www.fil.ion.ucl.ac.uk/spm). The first 10 volumes for each subject were discarded to allow the signal to reach equilibrium and the participants to adapt to the scanning noise. The remaining volumes were corrected for the acquisition time delay between slices. Next, realignment was performed to correct the motion between time points. All of the subjects’ fMRI data were within the defined motion thresholds (i.e., translational or rotational motion parameters less than 2 mm or 2°). We also calculated frame-wise displacement (FD), which indexes the volume-to-volume changes in head position. There were no significant group differences in FD (*t* = 0.56, *P* = 0.58) between the patients (0.117 ± 0.007) and controls (0.113 ± 0.006). Several nuisance covariates (six motion parameters, their first-time derivations, and average BOLD signals of the ventricular and white matter) were regressed out from the data. Recent studies have reported that the signal spike caused by head motion significantly contaminated the final resting-state fMRI results even after regressing out the linear motion parameters (Power et al. 2012). Therefore, we further regressed out spike volumes when the FD of the specific volume exceeded 0.5. The datasets were then band-pass filtered in a frequency range of 0.01 to 0.08 Hz. In the normalization step, individual structural images were linearly co-registered with the mean functional image; the transformed structural images were segmented into gray matter, white matter, and cerebrospinal fluid. The gray matter maps were non-linearly transformed to the tissue probability maps in the Montreal Neurological Institute (MNI) space. Finally, each filtered functional volume was spatially normalized to the MNI space using the parameters estimated during the non-linear transformation and resampled into a 3-mm cubic voxel.

### rsFCD calculation and analysis

The rsFCD of each voxel was calculated using an in-house script that was written in the Linux platform according to the method described by Tomasi and Volkow (Tomasi and Volkow 2010, 2011a, b). Pearson’s linear correlation evaluated the strength of the functional connectivity between every pair of voxels in the entire brain. Two voxels with a correlation coefficient, which was larger than a predefined threshold, were considered to be significantly connected. To obtain reliable and robust results, we used multiple threshold levels (*R* = 0.4, 0.6 and 0.8) to compute the rsFCD. The rsFCD calculation was restricted to a whole brain mask including the cerebellum. The rsFCD at a given voxel x_0_ was computed as the total functional connectivity, k(x_0_), between x_0_ and all other voxels. This calculation was repeated for all voxels within the whole brain mask. To increase the normality of the distribution, grand mean scaling was performed by dividing the rsFCD of each voxel by the mean value of the whole brain. Finally, the rsFCD maps were spatially smoothed using a 6 mm × 6 mm × 6 mm full-width at half maximum (FWHM) Gaussian kernel.

Inter-group differences in rsFCD were voxel-wisely compared within a cerebellar mask using a general linear model with age and sex as nuisance variables. Correction for multiple comparisons was performed using the voxel-level false discovery rate (FDR) method with a corrected threshold of *P* < 0.05. The significant regions in 3 comparisons (corresponding to the three thresholds) were overlapped. The overlapping areas were identified as the regions of interest (ROIs) for the correlation analyses and were defined as seed regions for the subsequent rsFC analyses. The ROI-based correlation analyses with the clinical variables, including antipsychotic dosages of chlorpromazine equivalents, illness duration and PANSS scores, were performed for the patient group using partial correlation analysis with age and sex as the nuisance covariates. For these correlation analyses, a significant threshold was set at *P* < 0.05.

### rsFC analysis

Most of the data preprocessing steps for the rsFC analysis were the same as the preprocessing steps for the rsFCD calculation. The only difference was that functional images were smoothed using a Gaussian kernel of 6 mm × 6 mm × 6 mm FWHM after normalization. For each subject, Pearson’s correlation coefficients between the mean time series of the seed region and that of each voxel in the whole brain was computed and converted to z-values using Fisher’s r-to-z transformation to improve the normality. Next, the individual’s z-values were entered into a random-effects one-sample *t*-test in a voxel-wise manner using SPM8. FDR correction with *P* < 0.05 was used to identify brain regions that showed significant positive correlations with the seed region. Next, a two-sample *t*-test was performed within the positive rs-FC mask to quantitatively compare the differences in rsFC of the seed region between schizophrenia patients and healthy controls. Multiple comparisons were corrected using the FDR method with a corrected threshold of *P* < 0.05. To test the association between rsFC and the clinical variables, voxel-wise multiple regression analysis was performed for the patient group within a mask constructed from the group differences between patients and controls. A threshold of *P* < 0.01 was considered significant.

## Results

### Demographic and clinical characteristics

The demographic and clinical characteristics of the subjects are summarized in Table 1. There were no significant differences in sex (χ^2^ = 1.35, *P* = 0.25) or age (*t* = 0.48, *P* = 0.63) between schizophrenia patients and healthy controls. Eighty-seven patients received atypical antipsychotic medications during the MRI examinations, and the remaining 8 patients had never received any medications. In schizophrenia patients, the mean antipsychotic dosage of chlorpromazine equivalents was 446.5 ± 341.6 mg/d; the mean duration of illness was 121.4 ± 92.8 months; the mean scores of the PANSS positive sub-scale, PANSS negative sub-scale and general psychopathology sub-scale were 17.1 ± 7.9, 20.3 ± 9.1 and 34.1 ± 10.8, respectively.

**Table 1:** Demographic and clinical characteristics of the schizophrenia patients and healthy controls

Characteristics	Schizophrenia patients	Healthy controls	Statistics	*P* value
Number of subjects	95	93		
Age (years)	33.6 (7.8)	33.0 (10.2)	*t* = 0.48	0.63
Sex (female/male)	41/54	48/45	χ^2^ = 1.35	0.25
Antipsychotic dosage (mg/d) (chlorpromazine equivalents)	446.5(341.6)	-		
Duration of illness (months)	121.4(92.8)	-		
PANSS				
Positive score	17.1(7.9)	-		
Negative score	20.3(9.1)	-		
General score	34.1(10.8)	-		
Total score	71.5(23.2)	-		

Data are shown as the means (SD). Abbreviations: PANSS, Positive and Negative Syndrome Scale

### Group difference in rsFCD

**Fig. 1:**
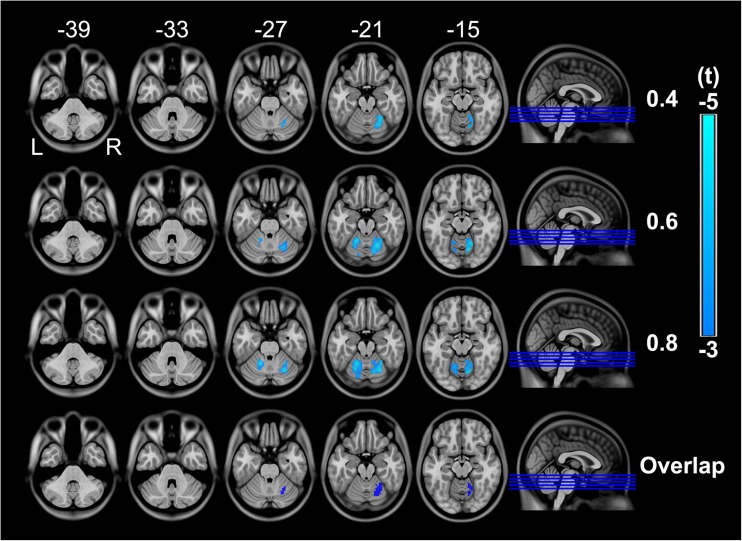
Intergroup differences in rsFCD with the correlation thresholds of 0.4, 0.6 and 0.8 (*P* < 0.05, FDR corrected) and their overlap. The cold color denotes decreased rsFCD in schizophrenia patients. L, left; R, right; rsFCD, resting-state functional connectivity density

At the correlation thresholds of *R* = 0.4, 0.6 and 0.8, the schizophrenia patients exhibited overlapping decreased rsFCD in the right hemispheric VI of the cerebellum compared with that of the healthy controls (*P* < 0.05, FDR corrected) (Fig. 1). No significant correlations were identified between the rsFCD in this region and clinical variables. Because 8 patients had never received any medications, we repeated the correlation analysis between rsFCD and antipsychotic dosage after excluding the 8 patients. Likewise, no significant correlation was found.

### Group difference in rsFC

**Fig. 2:**
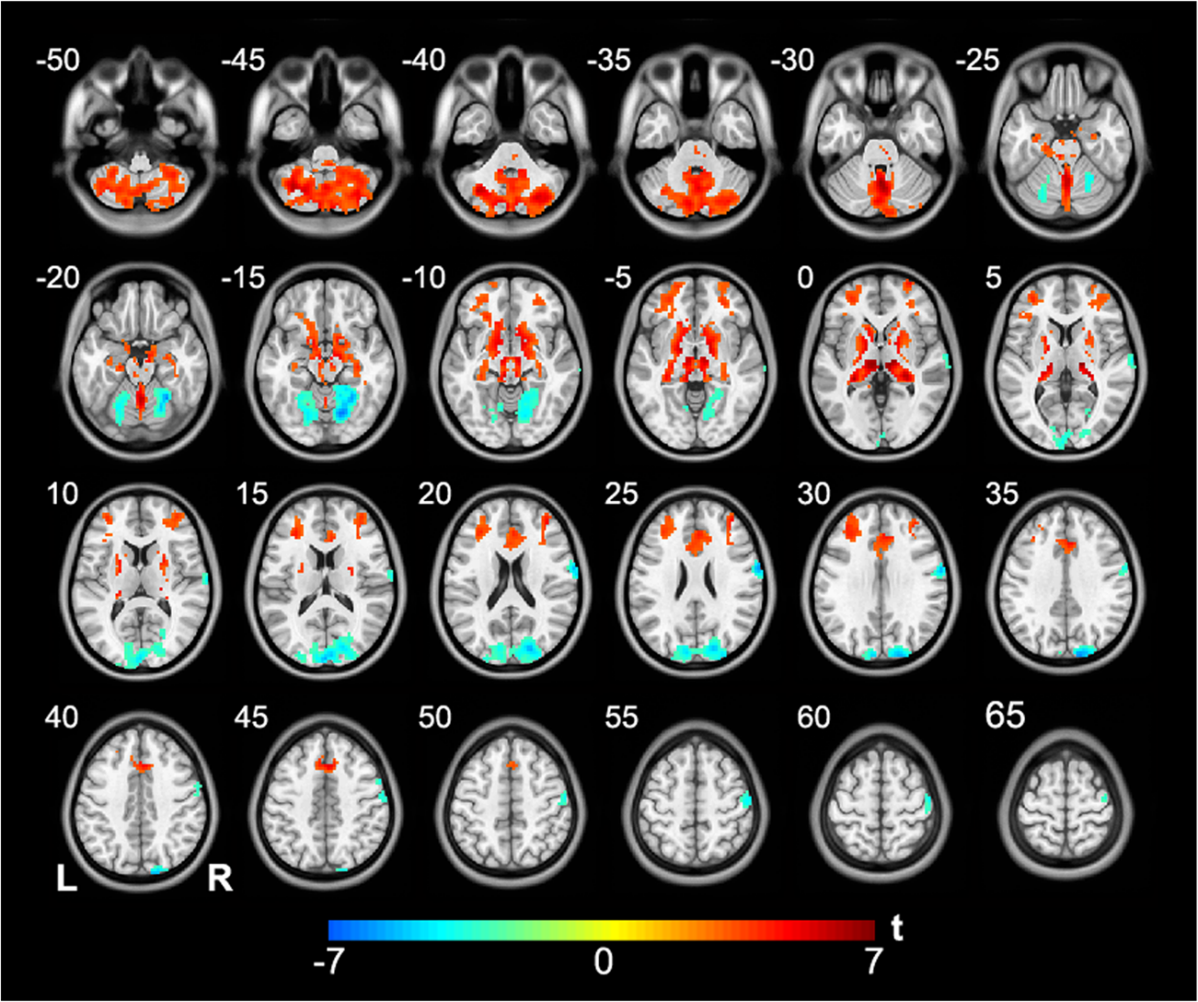
Brain regions showing altered rsFC with the cerebellar seed in schizophrenia patients. The warm color represents increased rsFC, and the cold color denotes decreased rsFC in schizophrenia patients. L, right; R, right; rsFC, resting-state functional connectivity

**Fig. 3:**
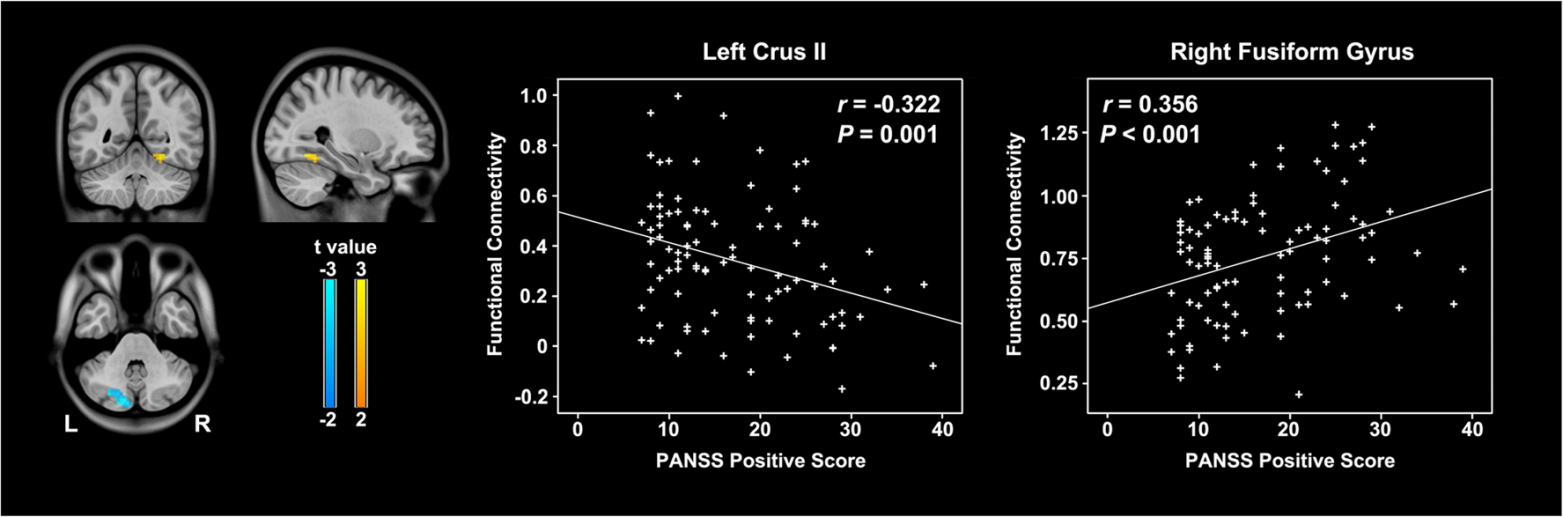
Correlations between altered rsFC and severity of the positive symptoms in schizophrenia patients. The warm color represents a positive correlation, and the cold color denotes a negative correlation. L, right; R, right; rsFC, resting-state functional connectivity; PANSS, Positive and Negative Syndrome Scale

Compared with the healthy controls, schizophrenia patients had increased rsFC between the right cerebellar VI and bilateral anterior cingulate cortex, dorsolateral prefrontal cortex, striatum, thalamus, and lower-middle part of the cerebellum and decreased rsFC between the right cerebellar VI and bilateral visual cortex and upper part of the cerebellum, and the right sensorimotor cortex (*P* < 0.05, FDR corrected) (Fig. 2). In schizophrenia patients, the rsFC between the right cerebellar VI and left cerebellar Crus II was negatively correlated with the PANSS positive score (Pearson’s correlation coefficient *r* = −0.322, *P* = 0.001); the rsFC between the right cerebellar VI and right fusiform gyrus was positively correlated with the PANSS positive score (*r* = 0.356, *P* < 0.001) (Fig. 3). Similar to the relationship between rsFCD and antipsychotic dosage, no significant correlation was found between rsFC and antipsychotic dosage in the present study.

## Discussion

In this study, we combined the rsFCD and rsFC methods to investigate the connectivity alterations of the cerebellum in schizophrenia. We found that schizophrenia patients exhibited decreased rsFCD in the right hemispheric VI; moreover, this cerebellar region showed increased rsFC with the prefrontal cortex and subcortical nuclei, and decreased rsFC with the visual cortex and sensorimotor cortex. In addition, some rsFC changes were associated with the positive symptoms. These findings suggest that the abnormality of the cerebellar functional connectivity may be a neural mechanism of schizophrenia.

The human cerebellum is a heterogeneous structure and has been anatomically divided into vermal and hemispheric subregions designated I-X(Schmahmann et al. 1999). Task-based neuroimaging studies have provided evidence that the cerebellar subregions are involved in multiple functions, including motor-related processing (Brown et al. 2006), cognitive and affective processing (Stoodley and Schmahmann 2010), pain-related processes (Dimitrova et al. 2004), and the experience of thirst (Parsons et al. 2000). A prior rsFC study has confirmed the existence of both functional integration and segregation in the cerebellum—that is, the functional integration is characterized by several subregions involved in the same functional network, whereas the functional segregation refers to different subregions involved in different functional networks (Sang et al. 2012). For example, hemispheric VI was correlated with the visual network, auditory network, sensorimotor network, salience network, and striatum, indicating that this cerebellar region is a functional hub that is of high connectivity to the cerebral cortex and subcortical nuclei (Sang et al. 2012). Using the rsFCD method that is sensitive in the detection of distribution alterations of functional hubs, we also found rsFCD abnormality in hemispheric VI in schizophrenia, suggesting functional connection number alterations of the cerebellar hub may be one important brain characteristic of schizophrenia.

To investigate the connection alterations that drove the rsFCD changes, we performed whole-brain rsFC analysis using hemispheric VI as the seed region. Both increased and decreased rsFC were identified in schizophrenia, although the combined effect was decreased rsFCD. Specifically, hemispheric VI exhibited increased rsFC with the prefrontal cortex and subcortical nuclei as well as decreased rsFC with the visual cortex and sensorimotor cortex, suggesting a disruption of the cerebellar-subcortical-cortical loop. Notably, the hyperconnectivity is mainly located in higher-order cerebral systems involved in cognitive and emotional processes, and the hypoconnectivity is predominantly located in lower order systems implicated in sensory processing and motor regulation, which may be used to guide future studies. In addition, we found a negative correlation between increased intra-cerebellar rsFC and the severity of the positive symptoms, and a positive correlation between decreased cerebellar-visual rsFC and the severity of positive symptoms. These findings suggest that mild rsFC abnormalities may lead to the development of positive symptoms, whereas excessive abnormality might preclude the formation of positive symptoms. Alternatively, the seemingly more “normal” rsFC may be the result of exposure to the more severe positive symptoms.

This study has several limitations. First, most of our patients had chronic schizophrenia and were receiving antipsychotic medications. Although we did not find any significant correlations between the altered rsFCD/FC and illness duration/antipsychotic dosages, we cannot absolutely rule out the effects of illness duration and antipsychotic medication on connectivity. In future studies, first-episode medication-naïve schizophrenia patients are needed to validate the findings of this study. Second, to calculate the rsFCD, a predefined correlation threshold must be set. However, there is no uniform standard for the selection of an appropriate threshold. In the present study, we used multiple thresholds (*R* = 0.4, 0.6 and 0.8) to compute the rsFCD and found that our main results were reproducible after considering the effects of different correlation thresholds. Third, because we were limited at that time by the lack of an ideal Chinese version cognition assessment tool specific to schizophrenia patients, such as the Consensus Cognitive Battery (MCCB) or the Cognitive Impairment in Psychiatry (SCIP) (Wu et al. 2016; Shi et al. 2015; Sánchez-Torres et al. 2016), we did not assess the schizophrenia patients’ cognition abilities and we did not investigate the relationship between rsFCD or rsFC alterations in the cerebellum in schizophrenia and cognition ability. We plan to explore it in a future study. Fourth, the lifetime antipsychotic dosage is more meaningful than the daily dosage to investigate the brain function activity alterations in schizophrenia patients, and we must collect the lifetime antipsychotic dosage information to analyze the influence of the antipsychotics to the functional activity of the brain. However, because many patients in the clinical setting cannot accurately recall their lifetime antipsychotic dosage because they had taken several antipsychotics in the past years, we could not collect their lifetime antipsychotic dosage in this study. We only recorded the daily antipsychotic dosage at the time of MRI scanning. We must consider the effect of the lifetime antipsychotic dosage in the future work to obtain more useful information regarding the mechanism of schizophrenia.

In conclusion, although some limitations exist, we revealed a disrupted functional connectivity pattern in the cerebellum in schizophrenia by the combination of rsFCD and rsFC methods. Specifically, we found decreased rsFCD in the right hemispheric VI and altered rsFC between this region and the cerebrum. These findings suggest that abnormalities of the cerebellar hub and cerebellar-subcortical-cortical loop may be the underlying mechanisms of schizophrenia.

### Author contributions

CZ and JZ designed the current study and wrote the paper. All of the authors performed the experiments and analyzed the data. All of the authors read and approved the final manuscript.
